# A challenging case of Cushing’s disease complicated with multiple thrombotic phenomena following trans-sphenoidal surgery; a case report

**DOI:** 10.1186/s12902-021-00701-0

**Published:** 2021-02-23

**Authors:** Piyumi Sachindra Alwis Wijewickrama, Vithiya Ratnasamy, Noel P. Somasundaram, Manilka Sumanatilleke, Sathyajith Buddhika Ambawatte

**Affiliations:** 1 Ministry of Health, Colombo, Sri Lanka; 2 National Hospital of Sri Lanka, Colombo, Sri Lanka

**Keywords:** Cushing’s disease, Thrombosis, Stroke, Diverticular rupture, Case report

## Abstract

**Background:**

Cushing’s syndrome occurs due to overproduction of cortisol from adrenal glands. Endogenous hypercortisolemia can occur secondary to adrenocorticotropic hormone (ACTH) dependent as well as independent causes. The presence of non-specific symptoms and signs contributes to a delay in diagnosis. Early identification and prompt definitive management is crucial. It is important to be alert about the post-operative complications including multiple thrombotic phenomena, which can add to the mortality. We report a case of Cushing’s disease in a young female managed with trans-sphenoidal surgery, followed by a challenging post-operative period complicated with multiple thrombotic phenomena, ultimately succumbed.

**Case presentation:**

A 32-year-old Sri Lankan female presented with overt features of Cushing’s syndrome and diagnosed to have ACTH dependent Cushing’s disease with pituitary microadenoma. She underwent trans-sphenoidal surgery, following which she developed fatal multiple complications including diverticular rupture and ischemic colitis, needing hemicolectomy, followed by a parieto-occipital infarction.

**Conclusion:**

This case highlights important and aggressive complications associated with Cushing’s syndrome giving rise to a challenging post-operative course. Diverticular rupture had been described in association with hypercortisolemia and this case adds to the existing literature. Post-operative ischemic colitis and stroke which contributed to the death of this patient could have been due to the procoagulant state associated with Cushing’s syndrome, with a high risk during the immediate post-operative period. This emphasizes the need to consider post-operative thromboprophylaxis in patients undergoing surgery for Cushing’s syndrome.

## Background

Cushing’s syndrome (CS) is caused by over production of cortisol from adrenal glands. This carries a very high morbidity and mortality due to associated complications if prompt diagnosis and effective management is not carried out.

Endogenous hypercortisolism is categorized as adrenocorticotropic hormone (ACTH) dependent and independent causes. ACTH dependent CS contributes to 80–85%, out of which Cushing’s disease (CD) due to pituitary ACTH hypersecretion is the most common, contributing to 75–80%, while 15–20% are due to ectopic ACTH syndrome [1]. ACTH independent CS due to adrenal adenomas or adrenal carcinomas contribute to 15–20% of the cases [1]. Usually, pituitary adenomas are microadenomas with only 5–10% of macroadenomas [2].

Patients present with a variety of clinical manifestations most of which are common with other diseases as well, such as obesity, hypertension, impaired glucose tolerance and menstrual irregularities, making the clinical diagnosis challenging. However, the presence of more discriminatory features, including purple striae, plethora, proximal myopathy, easy bruising, thin skin and unexplained osteoporosis should prompt the clinician to evaluate further with initial investigations including urinary free cortisol, late night salivary cortisol, 1 mg overnight dexamethasone suppression test (ODST), and standard two-days 2 mg dexamethasone suppression test (LDDST) [1, 3].

Once the diagnosis is achieved, further investigations should be carried out to establish the cause. Plasma ACTH can distinguish between ACTH dependent and ACTH independent causes. Magnetic resonance imaging (MRI) of pituitary, followed by inferior petrosal sinus sampling (IPSS) if necessary is warranted for further evaluation of ACTH dependent CS [1, 4].

It is of utmost importance to promptly control the disease and proceed with definitive management in order to minimize the associated morbidity and mortality. Cardiovascular diseases, cerebrovascular events and infections are the key contributors to mortality associated with CS [5]. The first line of management of CD is the excision of pituitary tumor, preferably via trans-sphenoidal approach, which is known to result in an initial remission rate of 60–80% [1]. However, effective management becomes extremely challenging due to multiple associated complications specially the thrombotic events which are more prominent in the immediate post-operative period, requiring close monitoring.

We report a case of CD in a young female due to ACTH secreting pituitary microadenoma managed with trans-sphenoidal surgery, with a challenging post-operative period due to multiple complications including thrombotic phenomena, ultimately succumbed.

## Case presentation

A 32-year-old Sri Lankan teacher, mother of one, presented with progressive weight gain and lower limb swelling for 6 months. She also noted gradual change in her appearance with hyperpigmentation, acne and facial swelling. She had lower limb proximal muscle weakness and noticed recent memory impairment over the last 6 months which significantly affected her teaching activities. Her mentation was normal without suicide ideas or psychosis.

Her menstruation had been regular until 3 month ago, after which she became amenorrhoeic and pregnancy was excluded. She also experienced loss of libido. She gave a history of intermittent, vague generalized headache for 6 months duration, without associated visual disturbances. There was no galactorrhea. There was no back pain or fractures.

She was diagnosed with primary hypothyroidism 10 years ago and she was on levothyroxine 100 μg by the time she presented to us. She did not have a chronic cough, wheezing or flushing episodes. There was no history of exogenous steroid intake.

She has a 3-year-old child and there was no history of adverse pregnancy outcomes including miscarriages. There was no family history of similar illnesses.

**Fig. 1:**
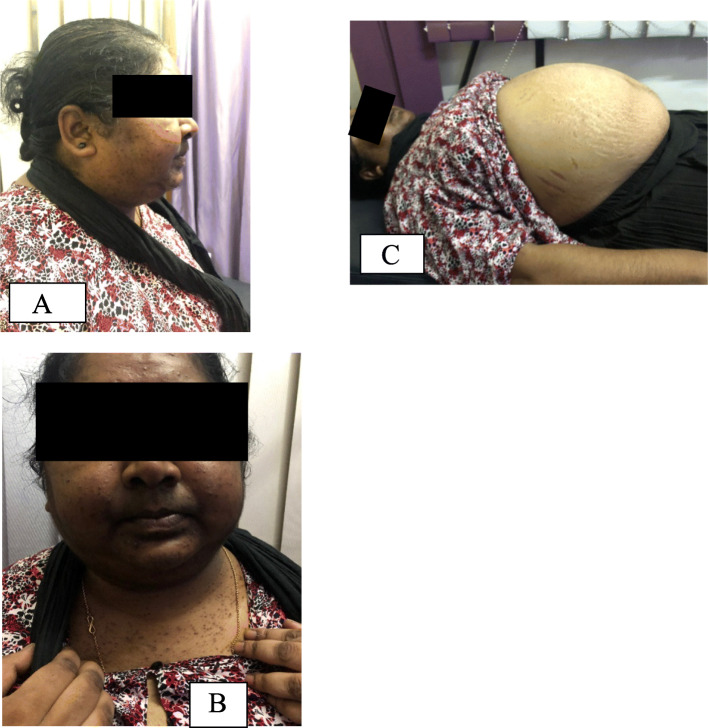
Examination findings supportive of Cushing's syndrome. **a** characteristic fat distribution in temporal region, cheeks and dorsal fat pad. **b** Facial plethora, hirsutism and acne. **c** Central obesity with purple striae over the abdomen

On examination, she was obese with body mass index (BMI) of 38 kg/m^2^, with predominant central obesity and peripheral wasting. Her face was plethoric and round, with fat accumulation in cheeks and temporal areas, as well as dorsocervical and supraclavicular fat deposition giving rise to a thick, short neck. She had acne, hirsutism mainly involving face and upper body, as well as acanthosis nigricans. She had thin skin, ecchymoses at the venipuncture sites, together with wide, purplish striae over her upper arms and abdomen (Fig. 1). She had hyperpigmentation mainly involving the face and nailbeds. She had bilateral symmetric pitting lower limb edema up to mid-calf level without any tenderness or redness. There was no goiter. Her pulse rate was 88 beats per minute and blood pressure was 150/100 mmHg. Her respiratory and abdominal examinations were normal. She had reduced proximal muscle power in both upper and lower limbs. Her visual field examination was normal, as well as the fundi.

All her clinical manifestations were suggestive of CS. This patient had characteristic fat distribution supportive of CS, as well as discriminatory features including purple striae, proximal muscle weakness and ecchymoses which are more predictive, together with young hypertension.

Her initial investigations are summarized in Table 1. Complete blood count revealed basophilia with low eosinophil count. Her potassium was low normal at 3.6 meq/L and she had high fasting blood glucose level, which further supported the diagnosis. Although the patient was initially treated for a primary hypothyroidism for 10 years, her current thyroid functions was suggestive of secondary hypothyroidism with inadequate thyroxine replacement, which is a common phenomenon seen in CS due to the suppression of thyroid stimulating hormone (TSH) pulse amplitude due to hypercortisolemia as well as due to blunted TSH response to thyroid releasing hormone [6].

**Table 1:** Summary of investigations

Investigation	Report	Normal range
White Blood Cell count	8.4 × 10^9^ /L	4–11 × 10^9^ /L
Neutrophils	78.7%	40–80%
Lymphocytes	14.6%	20–40%
Basophils	0.9%	0.1–0.2%
Eosinophils	0.8%	1–6%
Haemoglobin	10.9 g/dL	11–14 g/dL
Platelet count	286 × 10^9^/L	150–400 × 10^9^/L
Fasting Blood Sugar	175 mg/dL	
Serum Sodium	136 meq/L	135–145 meq/L
Serum potassium	3.6 meq/L	3.5–5.3 meq/L
Serum creatinine	0.8 mg/dL	0.6–1.1 mg/dL
AST	19.8 u/L	10–40 u/L
ALT	57 u/L	7–56 u/L
Thyroid Stimulating Hormone (TSH)	0.78 mIU/L	0.55–4.78 mIU/L
Free thyroxine level	0.9 ng/dL	0.89–1.76 ng/dL
Serum 9 am cortisol (Base line)	1147 nmol/L	118.6–618 nmol/L
Overnight dexamethasone suppression test	728 nmol/L	< 50 nmol/L
Serum adrenocorticotrophic hormone level	318 pg/mL	0–46 pg/mL
High dose dexamethasone suppression test	248 nmol/L	
Follicle Stimulating Hormone	5.27 IU/L	1.5–9.1 IU/L
Luteinizing Hormone	1.82 IU/L	0.5–16.9 IU/L
Serum Prolactin	193 mIU/L	59–619 IU/L

Her ODST was positive at 728 nmol/L, suggestive of CS. ACTH was very high at 318 pg/mL, suggestive of ACTH dependent CS. High dose dexamethasone suppression test (HDDST) was 248 nmol/L showing a 50% suppression compared to baseline, indicating the likelihood of CD. Contrast enhanced computed tomography (CT) chest, abdomen and pelvis which was done to look for an ectopic source was negative apart from bilateral adrenal hyperplasia.

**Fig. 2:**
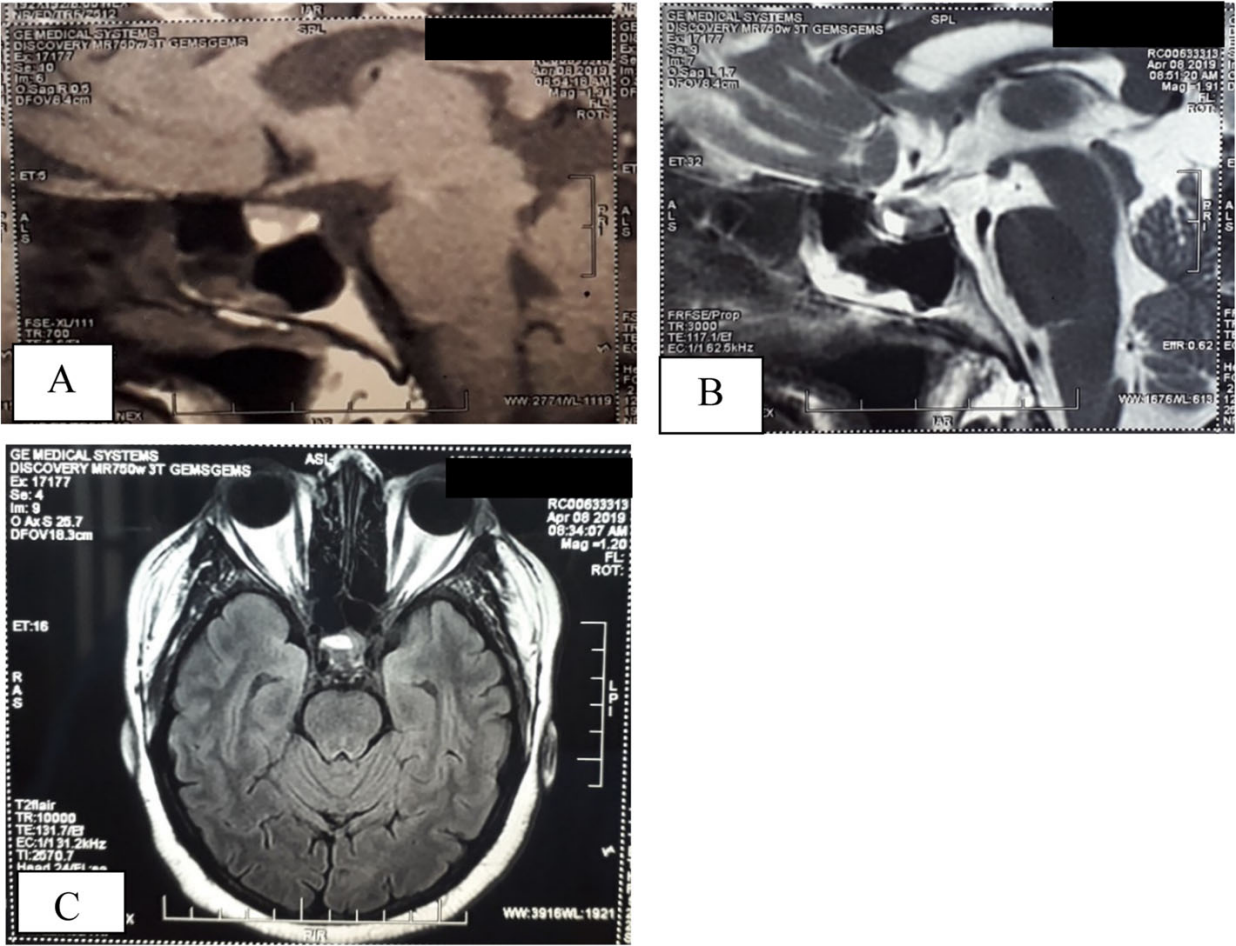
MRI showing high signal lesion of 10x8x9 mm suggestive of pituitary microadenoma. **a** T1 contrast sagittal. **b** T2 sagittal. **c** FLAIR axial

The MRI of pituitary revealed a high signal lesion of 10x8x9 mm in T1, T2 and FLAIR in the right side of pituitary, compatible with pituitary microadenoma (Fig. 2). IPSS revealed clear centralization and lateralization with a basal central to peripheral plasma ACTH ratio more than 2 and right side to left side inter sinus ratio of more than 1.4, confirming the source to be from right side of the pituitary (Table 2). Corticotrophin releasing hormone stimulation was not done due to unavailability of the reagent in local setting.

**Table 2:** Inferior Petrosal Sinus Sampling result – showing centralization and lateralization to right side

	Site	ACTH (pg/mL)	Cortisol (nmol/L)	Prolactin (mU/L)
1	Femoral	89.3	912.5	159.3
2	Left inferior petrosal sinus	213	1016.7	154.2
3	Right inferior petrosal sinus	721.3	938.5	359.1
4	Left internal jugular vein	166	987.7	170.4
5	Right internal jugular vein	234	1033.9	174.6

The patient was diagnosed with CD secondary to pituitary microadenoma. A trans-sphenoidal surgery was planned. Her disease was medically managed with ketoconazole 200 mg twice daily, with gradually increasing doses up to 400 mg twice daily, until the surgery, for 1 month. Controlling her CD burden was found to be extremely difficult, especially as ketoconazole was the only available medication at our setting which could be used for medical management of CS. Supportive management was done with anti-hypertensives, oral hypoglycemic drugs, and potassium supplements.

Within 1 month of diagnosis, she underwent endoscopic transnasal transsphenoidal hypophysectomy. Pituitary tumor was identified and removed by ring curettage and suction. Histology revealed pituitary adenoma with Ki67 proliferation index less than 1%.

Post- operatively, the patient was started on intravenous (IV) Hydrocortisone replacement of 50 mg 6 hourly, while continuing her thyroxine replacement. Serum 9 am cortisol on post-operative day 2 after withholding Hydrocortisone for 12 h came down to 80 nmol/L, indicating remission following surgery.

On post-operative day 5, patient complained of severe generalized abdominal pain. She had no associated vomiting and had opened bowel. On examination, her abdomen was distended, with generalized tenderness, without any palpable masses and intact bowel sounds. Patient had fever with temperature up to 102 degrees Fahrenheit. Lower limb examination did not reveal features suggestive of deep vein thrombosis. Her potassium was kept within the normal range with potassium supplements. She had neutrophilic leukocytosis with elevated C-reactive protein (CRP) at 56 mg/L. She was started on empirical IV antibiotics. However, she continued to have fever with CRP rising to 160 mg/L and continued to deteriorate with repeated X rays suggesting bowel perforation. Patient underwent an immediate explorative laparotomy, which revealed cecal perforation, after which right hemicolectomy with ileostomy and colostomy was done. Her clinical condition improved after the surgery.

Histology of right hemicolectomy specimen revealed cecal perforation with ruptured diverticulum, areas of hemorrhages, purulent material on serosal surface, thinned out, erythematous cecal wall with attenuated muscle wall and edematous submucosa. These morphological features were supportive of a ruptured diverticulum associated with serositis and ischemic colitis. The rest of the colonic mucosa was unremarkable without other diverticulae, crypt abscesses, dysplasia or malignancy.

**Fig. 3:**
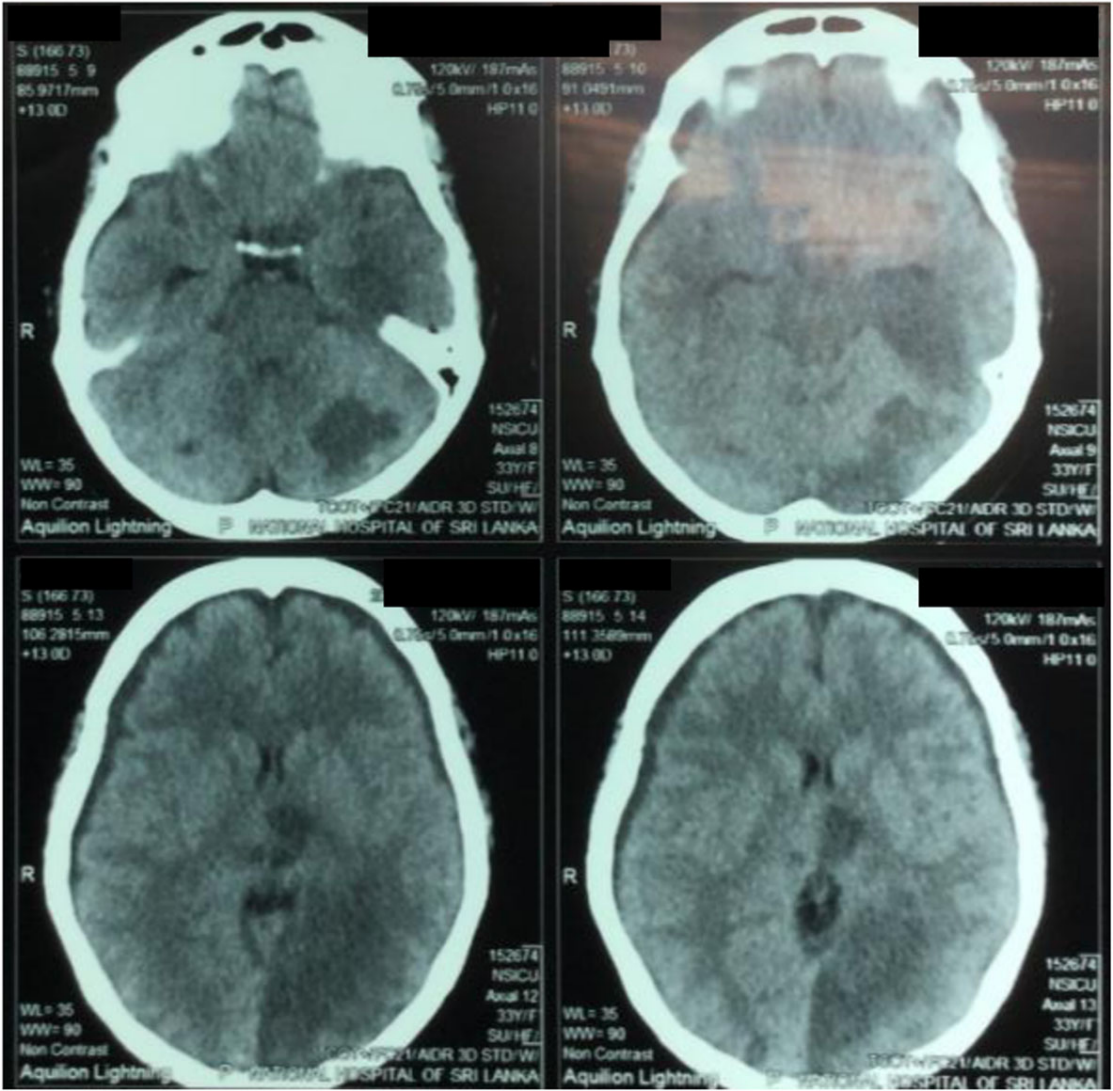
NCCT brain showing left sided parieto-occipital infarction, with involvement of the cerebellum

Thereafter, on post-operative day 14 following the pituitary surgery, while the patient was being monitored in the intensive care unit, her conscious level suddenly deteriorated down to Glasgow Coma Scale (GCS) 7/15, and she was intubated and ventilated. The non-contrast CT (NCCT) brain revealed left parieto-occipital and cerebellar infarctions (Fig. 3). The patient continued to deteriorate, with further progression of infarcted area, and died 24 h later despite all attempts of resuscitation.

## Discussion & conclusions

This case highlights the severity of complications associated with CD and that complications can even occur after surgical remission during immediate post-operative period following a prolonged disease course with poorly controlled hypercortisolemia.

The diagnosis of CD in this patient was straightforward and surgery was planned as the definitive therapy. Until the surgery, her disease was controlled with ketoconazole, a steroidogenesis inhibitor which is known to have a median response rate of 64% [7].

Following the trans-sphenoidal surgery her post-operative period became complicated despite achieving remission, with bowel perforation needing immediate laparotomy and hemicolectomy. Ischemic colitis with ruptured diverticulum were the most remarkable findings of the surgical specimens. Diverticular disease is widespread especially in the western countries. Usually, perforation of a diverticulum can occur due to high intracolonic pressure, disruption of colonic mucosal barrier, altered microflora and immunosuppression [8]. There is a well-known association between the treatment with exogenous corticosteroids and intestinal perforation. It was also previously observed that there is an association between intestinal perforation and endogenous glucocorticoid excess due to ACTH dependent CS [9, 10]. Interestingly, most of these patients did not have a history of pre-existing diverticular disease. Several mechanisms are responsible for the diverticular perforation associated with hypercortisolism, including reduced collagen turnover leading to weakened colonic wall integrity, reduction of prostacyclin formation and impaired wound healing. Glucocorticoid-induced activation of tumour necrosis factor alpha receptors may play a role [9]. Cortisol excess is also known to cause a delay in diagnosis of bowel perforation by masking typical symptoms. In all the patients who were studied, it was evident that the intestinal perforation occurred when they were hypercortisolemic and not when they were in remission. In contrast, our patient developed this intestinal perforation with diverticular rupture following the pituitary surgery when biochemically proven to be in remission, suggesting a possibility of the impact from long-lasting excess cortisol prior to achieving remission. The prolonged disease course prior to surgery with severe hypercortisolemia could have contributed to this.

Features of ischemic colitis were also present in the hemicolectomy specimen. This can be attributed to the thrombotic tendency seen in patients with CS, which has manifested as mesenteric ischemia. Thromboembolic complications are four-fold higher among patients with CS [11]. This is due to increased synthesis of fibrinogen and von Willebrand factor stimulated by cortisol, as well as increased synthesis of plasminogen activator inhibitor type 1 [12, 13]. Venous thrombo-embolism is the most commonly reported thrombotic phenomenon in these patients while acute mesenteric ischemia as seen in this patient is only rarely reported [14].

This patient also developed a left parieto-occipital stroke post-operatively, which may have largely contributed to her death. Cerebrovascular accident is a well-known complication in patients with CS, possibly contributed by the increased metabolic disease risk as well as procoagulant state. Patients with CS are known to be at high risk of stroke even before the diagnosis and the risk is known to remain elevated in long term follow up [15]. Vascular disease has been identified as the most common cause for death among patients with CS [5]. Furthermore, the involvement of multiple arterial territories suggests a possibility of underlying thromboembolic phenomena.

Clear guidelines do not exist regarding thromboprophylaxis in patients with CS. The Endocrine Society Clinical Practice guidelines suggest considering anticoagulation treatment peri-operatively specially as the risk of thrombo-embolism is highest in the first 4 weeks after surgery, due to worsening of the clotting profile [16, 17]. The sudden reduction of cortisol level with its anti-inflammatory activity, leading to increased risk of inflammation and thrombotic state also contributes to the post-surgical worsening of the thrombotic risk [12]. This could have been a contributory factor for these complications in our patient, as she had normal post-operative cortisol level.

In a retrospective analysis of 313 patients with CS, it was found that, before the introduction of prophylactic anti-coagulation, 10% of patients with CS died due to thrombo-embolism and 10% had vascular morbidity, while the introduction of prophylactic anticoagulation reduced the morbidity due to thromboembolic events to 6% and mortality to 0.4% [13]. Therefore, it is rational to treat these patients with thromboprophylaxis in the immediate post-operative period where the thrombo-embolic risk will be highest due to reduced ambulation, as well as drastic drop of cortisol level leading to a pro-inflammatory and a pro-thrombotic state [18].

Routine thromboprophylaxis for patients with CS had not been practiced in our center up to now. While further studies are needed to assess its benefits and risks, patient selection and the exact duration, post-operative thrombo-prophylaxis should be considered in patients with CS following surgery, especially in the presence of additional risk factors.

Overall, the time taken for diagnosis and attaining remission after the onset of symptoms have been identified as key factors which increase mortality in patients with CS suggesting that duration of hypercortisolemia is linked to increased mortality [19].

In conclusion, patients with CS are prone to multiple complications, contributing to increased mortality, out of which thrombo-embolic phenomena, especially during immediate post-operative period are well known. This case highlights the rare occurrence of mesenteric ischemia, diverticular rupture and stroke, complicating the post-operative period in a patient with CD, ultimately leading to death. Therefore, this emphasizes the need of the prompt diagnosis and treatment of CS, as well as the need for prophylactic anticoagulation in patients undergoing surgery for CS.

## Data Availability

The data used and/or analyzed during the current study are available from the corresponding author on reasonable request.

## Abbreviations

ACTH: Adrenocorticotropic hormone
CS: Cushing's syndrome
CD: Cushing's disease
ODST: Overnight dexamethasone suppression test
LDDST: Low dose dexamethasone suppression test
MRI: Magnetic Resonance Imaging
IPSS: Inferior petrosal sinus sampling
BMI: Body mass index
FBG: Fasting blood glucose
TSH: Thyroid stimulating hormone
TRH: Thyroid releasing hormone
HDDST: High dose dexamethasone suppression test
CRH: Corticotrophin releasing hormone
IV: Intravenous
CRP: C-reactive protein
GCS: Glasgow Coma Scale
NCCT: Non-contrast CT
